# Complete Genomes of Three Pseudomonas aeruginosa Bacteriophages, Kara-mokiny 1, Kara-mokiny 2, and Kara-mokiny 3

**DOI:** 10.1128/mra.00955-22

**Published:** 2022-11-07

**Authors:** Renee N. Ng, Anna S. Tai, Barbara J. Chang, Stephen M. Stick, Patricia Agudelo-Romero, Anthony Kicic, WAERP, AREST CF

**Affiliations:** a School of Biomedical Sciences, University of Western Australia, Perth, Western Australia, Australia; b Wal-Yan Respiratory Research Centre, Telethon Kids Institute, The University of Western Australia, Perth, Western Australia, Australia; c Department of Respiratory Medicine, Sir Charles Gairdner Hospital, Nedlands, Western Australia, Australia; d Institute for Respiratory Health, Nedlands, Western Australia, Australia; e Faculty of Health and Medical Sciences, The University of Western Australia, Perth, Western Australia, Australia; f The Marshall Centre for Infectious Diseases Research and Training, School of Biomedical Sciences, The University of Western Australia, Perth, Western Australia, Australia; g Department of Respiratory and Sleep Medicine, Perth Children’s Hospital, Nedlands, Western Australia, Australia; h Centre for Cell Therapy and Regenerative Medicine, School of Medicine and Pharmacology, The University of Western Australia and Harry Perkins Institute of Medical Research, Perth, Western Australia, Australia; i Australian Research Council Centre of Excellence in Plant Energy Biology, School of Molecular Sciences, The University of Western Australia, Perth, WA, Australia; j European Virus Bioinformatics Center, Jena, TH, Germany; k Occupation, Environment and Safety, School of Population Health, Curtin University, Perth, Western Australia, Australia; Queens College CUNY

## Abstract

Here, we present the complete genome sequence of Pseudomonas aeruginosa phages Kara-mokiny 1, Kara-mokiny 2, and Kara-mokiny 3. These phages have lytic capabilities against P. aeruginosa and belong to the myovirus morphotype. The genomes of Kara-mokiny 1 and Kara-mokiny 2 are 67,075 bp while that of Kara-mokiny 3 is 66,019 bp long.

## ANNOUNCEMENT

Pseudomonas aeruginosa has been identified by the World Health Organization (WHO) as a priority pathogen critically requiring new antimicrobials (1, 2). With multidrug resistance (MDR) complicating treatment regimens (3, 4), alternatives such as phage therapy are being explored (5–8).

P. aeruginosa phages Kara-mokiny 1, Kara-mokiny 2, and Kara-mokiny 3 were isolated from 2 freshwater ponds across the Perth metropolitan area (Western Australia, Australia, −31.961489406200172, 115.93334774661672 and −31.978662664328002, 115.94961261581557). Isolation involved filtering water samples through 0.22-μm filters and incubation with a propagating host, i.e., PAO1 (ATCC 15692), for 24 h at 37°C and 50 rpm. Cultures were then centrifuged and passed through a 0.22-μm filter, three rounds of plaque purification were performed, and cultures were stored at 4°C (9, 10).

Phage suspensions were then treated with DNase (Life Technologies, Australia) and RNase (Qiagen, Australia) to remove residual bacterial genome while the phage capsid was digested with proteinase K (Qiagen, Australia). DNA extraction and purification were then performed using a DNeasy Blood & Tissue kit as described by the manufacturer (Qiagen, Australia) (11, 12). The phage DNA library was generated using the Nextera XT preparation kit (Illumina Inc., USA) at the Australian Genome Research Facility (AGRF) in Melbourne, Australia. Libraries were sequenced on the Illumina MiSeq platform (Illumina, USA), which produced 150-bp paired-end (PE) reads.

Kara-mokiny 1 had 1,079,083 raw reads, Kara-mokiny 2 had 1,103,206 raw reads, and Kara-mokiny 3 had 1,274,579 raw reads, whose quality was checked via FastQC (www.bioinformatics.babraham.ac.uk/projects/fastqc). Adaptors were trimmed using BBDuk (B. Bushnell, https://sourceforge.net/projects/bbmap/) prior to assembly. Genome assemblies were conducted using Unicycler v0.4.9b (13), utilizing SPAdes for *de novo* assembly, resulting in one circular contig for each phage. Depth coverage was performed using BBMap (B. Bushnell, https://sourceforge.net/projects/bbmap/) and SAMtools (14), and assembled genomes were annotated using Prokka 1.13 (15) and the Prokaryotic Virus Remote Homologous Groups (PHROGS) database (16) with the HMM profile-profile comparisons. Antibiotic resistance (ResFinder [17], CARD [18], and ARG-ANNOT [19]) and bacterial virulence genes (VFDB [20]) were identified using available databases in ABRicate v1.0.1 (T. Seemann, ABRicate, https://github.com/tseemann/abricate). No tRNA was found in any of the phages using tRNAscan-SE v2.0 (21–23). Default parameters were used for all software unless otherwise specified.

Phage characteristics are shown in Table 1. Kara-mokiny 1, 2, and 3 were found to have 36 predicted phage-related proteins including one endolysin. A Basic Local Alignment Search Tool (BLAStn) search revealed Pseudomonas phage phiKTN6 (GenBank accession number NC_041865.1) to be the most closely related to Kara-mokiny 1 with an average nucleotide identity (ANI) of 97.52%. Kara-mokiny 2 was most closely related to Pseudomonas phage S50 (GenBank accession number LC472884.1) with 98.27% ANI, and Kara-mokiny 3 was most closely related to Pseudomonas phage 14-1 (GenBank accession number FM897211.1) with 96.59% ANI. Kara-mokiny 1, Kara-mokiny 2, and Kara-mokiny 3 are predicted to be of the myovirus morphotype in the class *Caudoviricetes*.

**TABLE 1:** Characteristics of phages

Phage name	Genome size (bp)	GC content (%)	Depth coverage (×)	No. of genes
Kara-mokiny 1	67,075	55.6	2,133.95	94
Kara-mokiny 2	67,075	55.6	2,533.02	95
Kara-mokiny 3	66,019	55.7	2,715.29	90

### Data availability.

The complete sequences of P. aeruginosa phages Kara-mokiny 1, Kara-mokiny 2, and Kara-mokiny 3 have been deposited in GenBank under accession numbers OP314870, OP314871, and OP314872, respectively, BioProject number PRJNA862988, and BioSample accession numbers SAMN30383118, SAMN30383119, and SAMN30383120, respectively.
